# Lithium overdosage and related tests

**DOI:** 10.1186/s40345-015-0044-y

**Published:** 2016-01-12

**Authors:** Paolo D. Pigatto, Bernardo Dell’Osso, Gianpaolo Guzzi

**Affiliations:** Department of Biomedical, Surgical and Dental Sciences, Unit of Oral Pathology and Medicine, IRCCS Galeazzi Hospital, University of Milan, Milan, Italy; Fondazione IRCCS Ca’ Granda Policlinico, Department of Pathophysiology and Transplant, University of Milan, Milan, Italy; Bipolar Disorders Clinic, Stanford Medical School, Palo Alto, CA USA; Italian Association for Metals and Biocompatibility Research-A.I.R.M.E.B., (not-for-profit organization), Via A. Banfi, 4, 20122 Milan, Italy

## Abstract

Lithium acts biochemically through the inositol depletion in brain cortex. At low doses, however, it is partly effective and/or ineffective, whereas in high concentrations is toxic. We would like to make one point about this review. In fact, in our view, the patient should be given a support to correct hypernatremia and even sodium levels should be tested serially—along with serum lithium concentrations—because high sodium levels reduce the rate of elimination of lithium. Lithium is mainly a neurotoxicant. Lithium-related central nervous system toxicity as well as the cardiovascular and thyroid changes are most likely due to the cations (Na_2_^+^ and K^+^) competition.

Haussmann and colleagues (2015) provide a very useful review of lithium intoxication. As they rightly point out, there is an urgent need for comprehensive evaluations in the clinical management of patients with lithium intoxication (Haussmann et al. 2015). Lithium, as lithium carbonate (Li_2_CO_3_) and/or lithium citrate (Li_2_C_6_H_5_O_7_), is among the most recommended drugs for the treatment of bipolar disorder by international guidelines (Casarett et al. 2008). Lithium acts biochemically through the inositol depletion in brain cortex (Casarett et al. 2008). At low doses, however, it is partly effective and/or ineffective, whereas in high concentrations is toxic (Casarett et al. 2008). Their study led the investigators to endorse a serial blood measurements of lithium (Haussmann et al. 2015) during the critical period of acute lithium poisoning (or acute-on-chronic intoxication), mentioning previous studies to support such a position (Timmer and Sands 1999; Casarett et al. 2008). Therefore, the authors suggest that measurement of lithium (i.e., every 2–4 h) (Haussmann et al. 2015) should be incorporated in medical management in the emergency department. However, we would like to make one point about this review. In fact, in our view, the patient should be given a support to correct hypernatremia and even sodium levels should be tested serially—along with serum lithium concentrations—because high sodium levels reduce the rate of elimination of lithium (Liamis et al. 2009; Grunfeld and Rossier 2009). Lithium is mainly a neurotoxicant (Casarett et al. 2008). Lithium-related central nervous system toxicity as well as the cardiovascular and thyroid changes are most likely due to the cations (Na_2_^+^ and K^+^) competition (Casarett et al. 2008). As the authors correctly assert, (Haussmann et al. 2015) pharmacologic management, laboratory testing, and specific recommendation should be implemented to improve effectiveness of lithium poisoning treatment.
